# Supplementary material for: Mycorrhiza-Tree-Herbivore Interactions: Alterations in Poplar Metabolome and Volatilome
Source: Metabolites. 2022 Jan 19;12(2):93. doi: 10.3390/metabo12020093 (PMC8880370; doi:10.3390/metabo12020093)
Supplement: Supplementary file 1 [file metabolites-12-00093-s001.zip › Supplementary Figures.pdf]

metabolites

MDPI

Supplementary Material

Mycorrhiza–Tree–Herbivore Interactions: Alterations in Poplar Metabolome and Volatilome

Prasath Balaji Sivaprakasam Padmanaban [1,†], Maaria Rosenkranz [1,†,*], Peiyuan Zhu [1], Moritz Kaling [1], Anna Schmidt [2], Philippe Schmitt-Kopplin [3], Andrea Polle [2] and Jörg-Peter Schnitzler [1,*]

Supplementary Figure S1

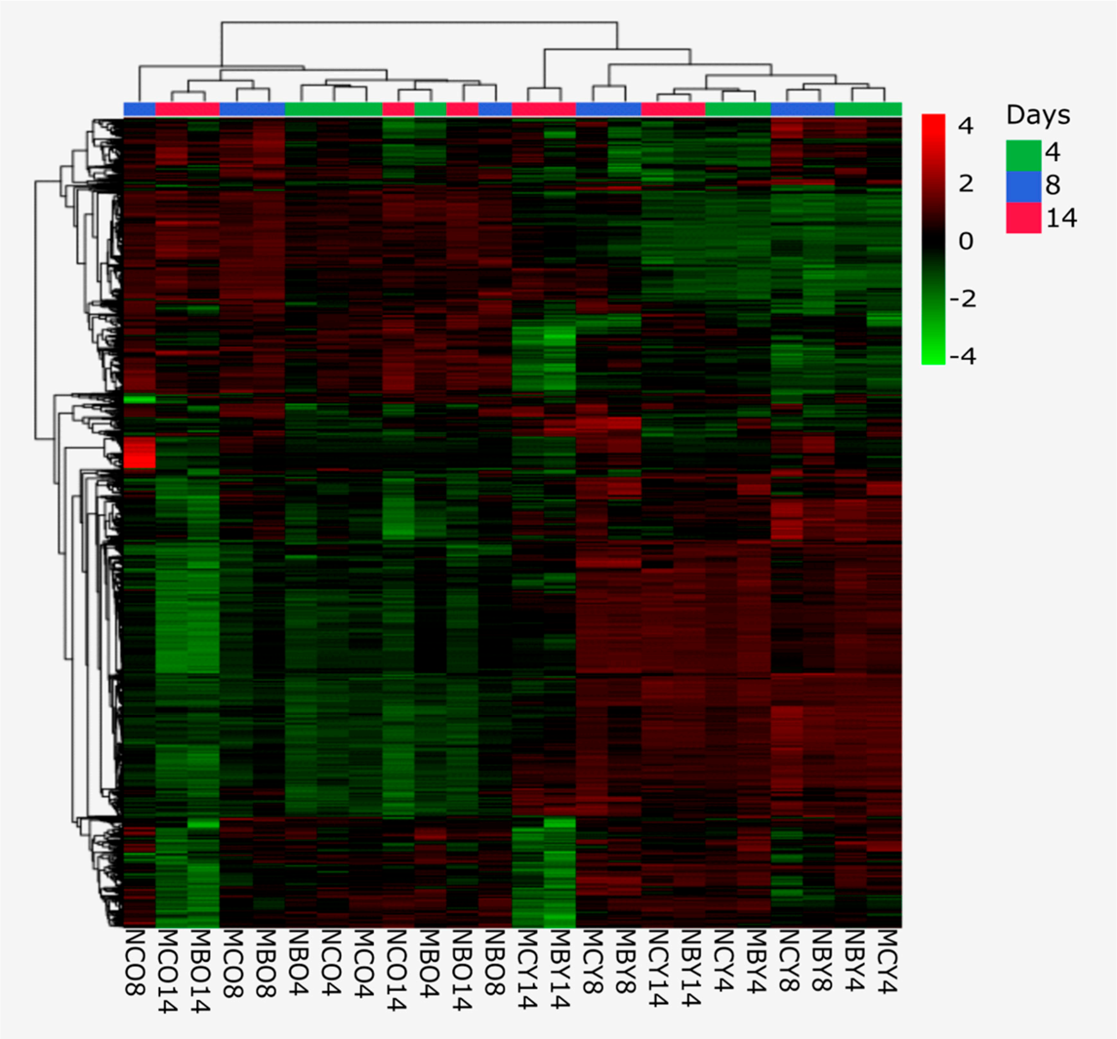

Supplementary Figure S1. Hierarchical clustering analysis (HCA with Euclidean distance) shows the LC-MS intensities of all mass features on the days 4, 8 and 14 after the release of herbivores. The data is shown for old (O) and young (Y) leaves of mycorrhized and non-mycorrhized poplar trees. NC = non-mycorrhizal poplars not exposed to leaf beetles, MC = mycorrhizal poplars not exposed to leaf beetles, NB = non-mycorrhizal poplars exposed to leaf beetles, and MB = mycorrhizal poplars exposed to leaf beetles.

Supplementary Figure S2

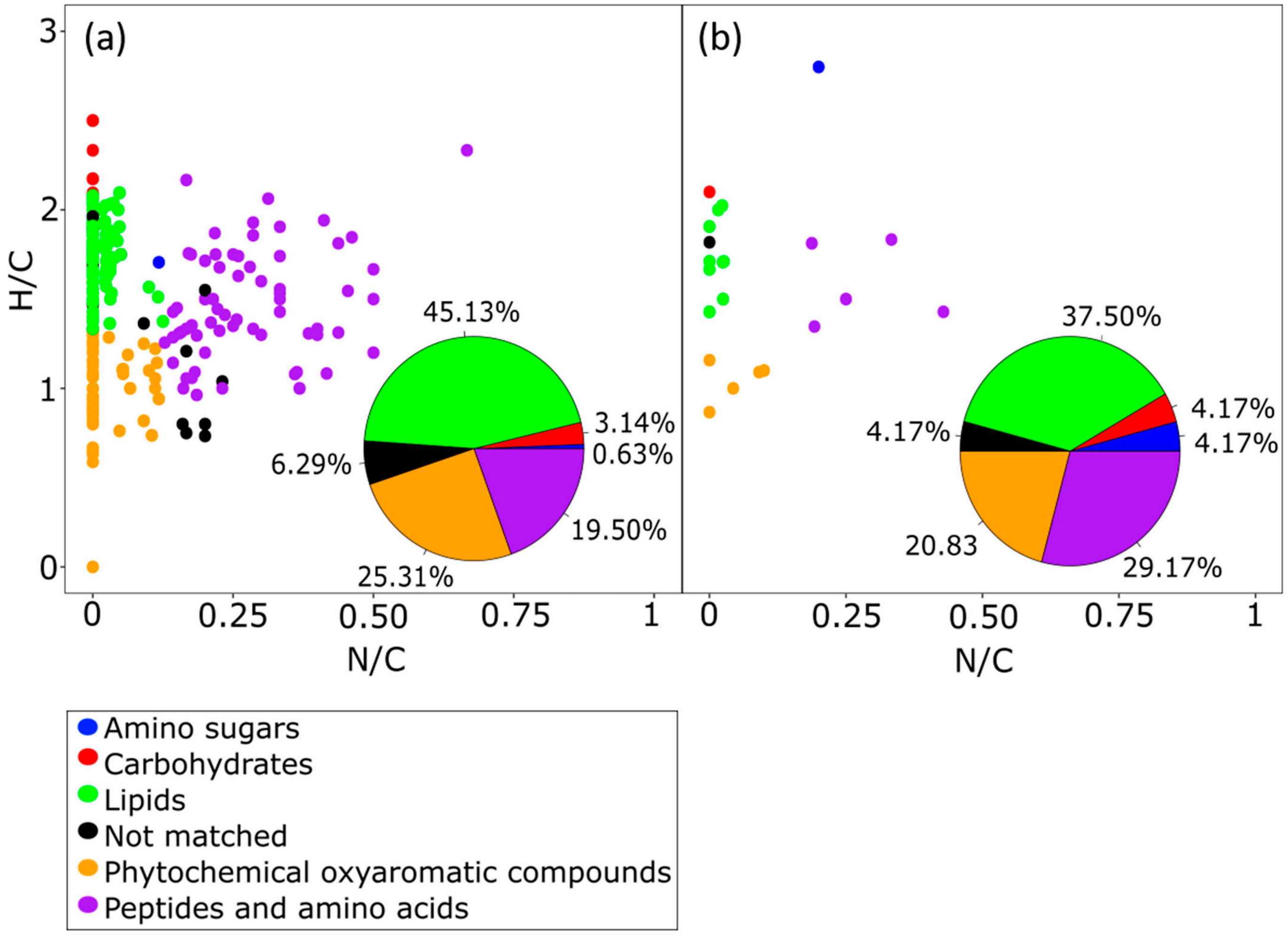

Supplementary Figure S2. Van Krevelen plot combined with multidimensional stoichiometric compound classification (MSCC) classifies all mass features according to C, H, N, O, S, and P content in leaves (a) and roots (b) of mycorrhized trees. The proportions of the classified mass features in each chemical group are given in the pie charts. All plotted mass features were discriminant for treatment (mycorrhization) separation in orthogonal partial least square regression discriminant analyses (OPLS-DA) (VIP > 1.0; CV-ANOVA < 0.05; individual mass feature *P*-values < 0.05; log2 (T/S) ratio of < −0.5 or > 0.5). Values are relative abundances, logarithmically transformed, and Pareto scaled with centering.
